# A SNARE protective pool antagonizes APOL1 renal toxicity in *Drosophila* nephrocytes

**DOI:** 10.1186/s13578-023-01147-8

**Published:** 2023-11-04

**Authors:** Jin-Gu Lee, Yulong Fu, Jun-yi Zhu, Pei Wen, Joyce van de Leemput, Patricio E. Ray, Zhe Han

**Affiliations:** 1 Center for Precision Disease Modeling, Department of Medicine, University of Maryland School of Medicine (UMSOM), 670 West Baltimore Street, 4052 HSFIII, Baltimore, MD 21201 USA; 2 Division of Endocrinology, Diabetes and Nutrition, Department of Medicine, University of Maryland School of Medicine, Baltimore, MD 21201 USA; 3 Child Health Research Center, Department of Pediatrics, University of Virginia School of Medicine, 409 Lane Road, Charlottesville, VA 22908 USA; 4 Department of Pathology, University of Alabama Birmingham, Birmingham, AL 35249 USA

**Keywords:** SNARE proteins, SNARE protective pool, APOL1, Renal toxicity, Nephrocytes, Serum resistance-associated (SRA) protein

## Abstract

**Background:**

People of Sub-Saharan African ancestry are at higher risk of developing chronic kidney disease (CKD), attributed to the *Apolipoprotein L1* (*APOL1*) gene risk alleles (RA) G1 and G2. The underlying mechanisms by which the APOL1-RA precipitate CKD remain elusive, hindering the development of potential treatments.

**Results:**

Using a *Drosophila* genetic modifier screen, we found that SNARE proteins (Syx7, Ykt6, and Syb) play an important role in preventing APOL1 cytotoxicity. Reducing the expression of these SNARE proteins significantly increased APOL1 cytotoxicity in fly nephrocytes, the equivalent of mammalian podocytes, whereas overexpression of *Syx7*, *Ykt6*, or *Syb* attenuated their toxicity in nephrocytes. These SNARE proteins bound to APOL1-G0 with higher affinity than APOL1-G1/G2, and attenuated APOL1-G0 cytotoxicity to a greater extent than either APOL1-RA.

**Conclusions:**

Using a *Drosophila* screen, we identified SNARE proteins (Syx7, Ykt6, and Syb) as antagonists of APOL1-induced cytotoxicity by directly binding APOL1. These data uncovered a new potential protective role for certain SNARE proteins in the pathogenesis of APOL1-CKD and provide novel therapeutic targets for *APOL1*-associated nephropathies.

## Background

**Fig. 1:**
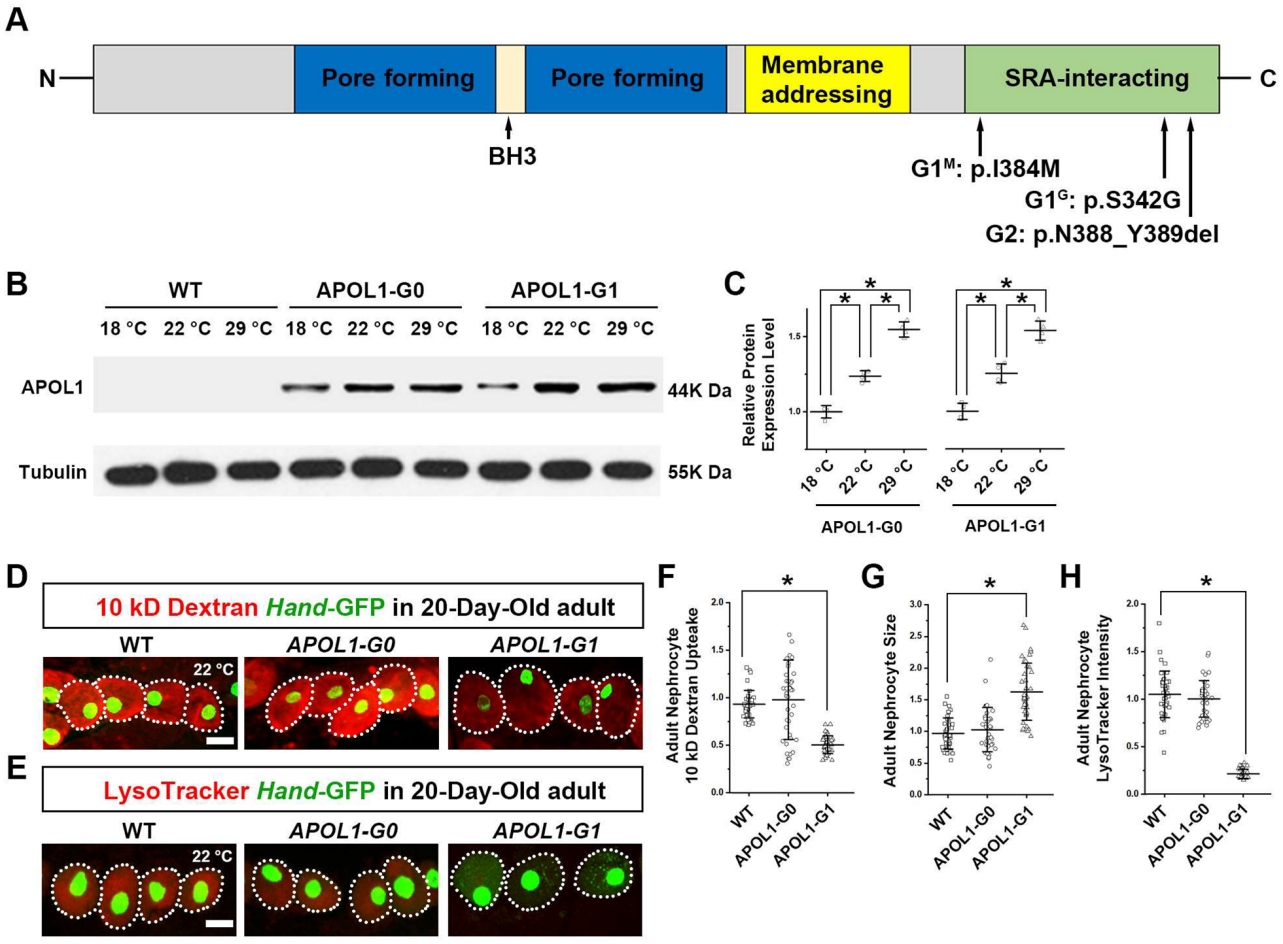
APOL1-induced nephrocyte phenotype is dose dependent. **A** Key APOL1 protein domains. Reference (*G0*) and risk allele (*G1* and *G2*) amino acid variants in the SRA-binding domain have been indicated. BH3, Bcl-2 Homology 3; SRA, serum resistance-associated protein. **B** Western blot analysis of APOL1 protein using anti-FLAG antibody. Total protein was extracted from third instar larvae carrying *Tub*-Gal4 to drive *APOL1-G0* and *APOL1-G1* expression; wildtype (WT) larvae carried *Tub*-Gal4 only. Tubulin was used as an internal reference. **C** Quantification of relative APOL1 protein level based on the western blot analysis in B. N = 10 larvae per sample; four independent blots. Data represented as mean ± SD. **D** Representative images of 10 kD fluorescent dextran particle uptake (red) by nephrocytes from 20-day-old adult flies using nephrocyte specific driver *Dot*-Gal4 to express *APOL1-G0* and *APOL1-G1* constructs, and wildtype (WT) larvae with *Dot*-Gal4 only, at 22 °C. *Hand*-GFP transgene expression was visualized as green fluorescence located in nephrocyte nuclei. Dotted lines outline the individual nephrocytes. **E** Representative images of LysoTracker (red) in nephrocytes from 20-day-old adult flies using nephrocyte specific driver *Dot*-Gal4 to express *APOL1-G0* and *APOL1-G1* constructs, and wildtype (WT) larvae with *Dot*-Gal4 only, at 22 °C. *Hand*-GFP transgene expression was visualized as green fluorescence located in nephrocyte nuclei. Dotted lines outline the individual nephrocytes. **F** Quantification of 10 kD dextran uptake in **D**, relative to wildtype (WT) value. N = 30 nephrocytes total obtained from 6 flies. **G** Quantification of adult nephrocyte size, relative to wildtype (WT) nephrocytes. N = 30 nephrocytes total obtained from 6 flies. **H** Quantification of LysoTracker signal in **E**, relative to wildtype (WT) value. N = 30 nephrocytes total obtained from 6 flies. Results represented as mean ± SD, statistical significance (*) P < 0.05

Millions of individuals of Sub-Saharan African ancestry face elevated risk of developing chronic kidney disease (CKD) associated with the inheritance of two common *Apolipoprotein L1* (*APOL1*) gene variant alleles (risk allele, or RA) designated *APOL1-G1* and *APOL1-G2* [1–3] (Fig. 1A). APOL1 provides immune protection against *Trypanosoma brucei*, a parasite native to Sub-Saharan Africa that causes African sleeping sickness [4]. *APOL1-RA* associated nephropathies include focal segmental glomerulosclerosis (FSGS), HIV-associated nephropathy (HIVAN), hypertension-associated end stage kidney disease (ESKD), sickle cell nephropathy, and lupus nephritis [2, 3, 5–8]. Despite intensive research, the mechanisms by which the *APOL1-RA* instigate *APOL1*-associated CKD are not clearly understood, and few specific treatments are currently available to prevent their progression [9]. Furthermore, transplanted kidneys from donors homozygous for *APOL1-RA* have shortened allograft survival in their recipients [10–12]. The development of effective treatment options for *APOL1*-associated nephropathies has been hampered by the lack of efficient animal model systems to study their pathogenesis and to perform reliable and cost-effective screenings for potential therapeutic targets.

The pathologic effects of APOL1-RA in the human kidney appear to follow a two-hit model, whereby a viral infection or inflammatory stimuli increases the expression level of APOL1 in podocytes and precipitates certain kidney diseases. Since the *APOL1* gene is only present in humans and certain primates, studying the mechanisms of *APOL1-RA* induced kidney toxicity often requires introducing *APOL1-RA* to either in vitro or in vivo models. As such, the effects of *APOL1-RA* have been studied extensively in cultured podocytes and renal epithelial cells [13–18]. However, these in vitro systems rely on the overexpression of *APOL1*, and do not reproduce the complex physiological conditions of podocytes in vivo. This approach is further complicated by the changes that occur in the intracellular localization of APOL1 when overexpressed, as well as the extreme sensitivity of cultured podocytes to this overexpression, such that even the non-risk (wildtype) APOL1-G0 allele may precipitate their death [13].

To overcome these experimental limitations, several transgenic *APOL1* mouse models have been developed using different experimental approaches [19–25]. In general, these models support the notion that the kidney disease is a function of the expression level of the *APOL1* in podocytes, and describe different mechanisms through which the risk variants may induce CKD (recently reviewed in detail [26]). One limitation of these transgenic mouse models is that they cannot be used in genetic screens to identify interacting proteins that modify the toxicity of APOL1. Therefore, we and others have generated *Drosophila APOL1* transgenic models to demonstrate that the expression of *APOL1-RA* in nephrocytes—fly cells with striking similarity to human podocytes—leads to cell death in vivo by affecting key intracellular trafficking pathways [27, 28]. These models were generated with the Gal4-UAS system to express the *APOL1* wildtype or risk alleles specifically in the nephrocyte [29]. *Drosophila* offers rapid access to genetic manipulation, thus enabling protein–protein interaction studies in a reliable, rapid, and cost-effective manner, when compared to mouse models.

In this study, we performed an unbiased genetic screening in *Drosophila* to identify genes that, when silenced in nephrocytes, could modify the toxicity of APOL1. We found that a protective pool of select SNARE (N-ethylmaleimide sensitive factor attachment protein receptors) proteins (*Syx7*, *Ykt6* and, *Syb*) play a critical role in this process, uncovering new potential therapeutic targets to prevent the progression of *APOL1*-associated nephropathies.

## Results

### Improving the *Drosophila* model to better recapitulate APOL1 nephropathy in patients

We previously generated transgenic *Drosophila* lines carrying human *APOL1-G0* or *APOL1-G1* with nephrocyte-specific expression (driven by Gal4; each line with the exact same docking site to ensure equal expression of the transgenes) [27]. The model was assayed at 29 °C, at which overexpression of non-risk *APOL1-G0* also caused cytotoxic effects, albeit milder than those induced by *APOL1-G1* overexpression [27]. Because the toxicity of APOL1 encoding transgenes appeared to be dose dependent and the stability of the Gal4 driver protein is temperature sensitive (Fig. 1B, C), we also tested flies reared at 22 °C or 18 °C. At 22 °C, we found that the toxic effects were nearly abolished in flies carrying the common non-risk APOL1-G0 allele (Fig. 1D–H). Whereas the toxic phenotype induced by the APOL1-G1 allele remained, including the previously reported reduced nephrocyte function (Fig. 1D, F), increased cell size (Fig. 1G), and decreased organelle acidification (Fig. 1E, H). Interestingly, under the new conditions (22 °C), no phenotype was detected in *APOL1-G0* flies at day 1. However, at day 20 these flies showed the beginnings of a toxic effect similar to what we observed on day 1 at 29 °C [27]. On the other hand, *APOL1-G1* flies already showed significant effects of toxicity at day 1 and these were even more pronounced at day 20 (Fig. 1D–H). These data confirm that in our fly model system the toxicity of APOL1 in nephrocytes is dose dependent. Moreover, *APOL1-G0* only showed toxicity, albeit mild, with no significant difference compared to wildtype flies until 30 days; *APOL1-G1* similarly showed increased severity over this period. These progressive patterns suggest possible accumulation of APOL1 over time which results in detectable toxicity once a threshold has been breached.

### Genetic screen reveals genetic modifiers of *APOL1-G1*-induced nephrocyte toxicity

**Fig. 2:**
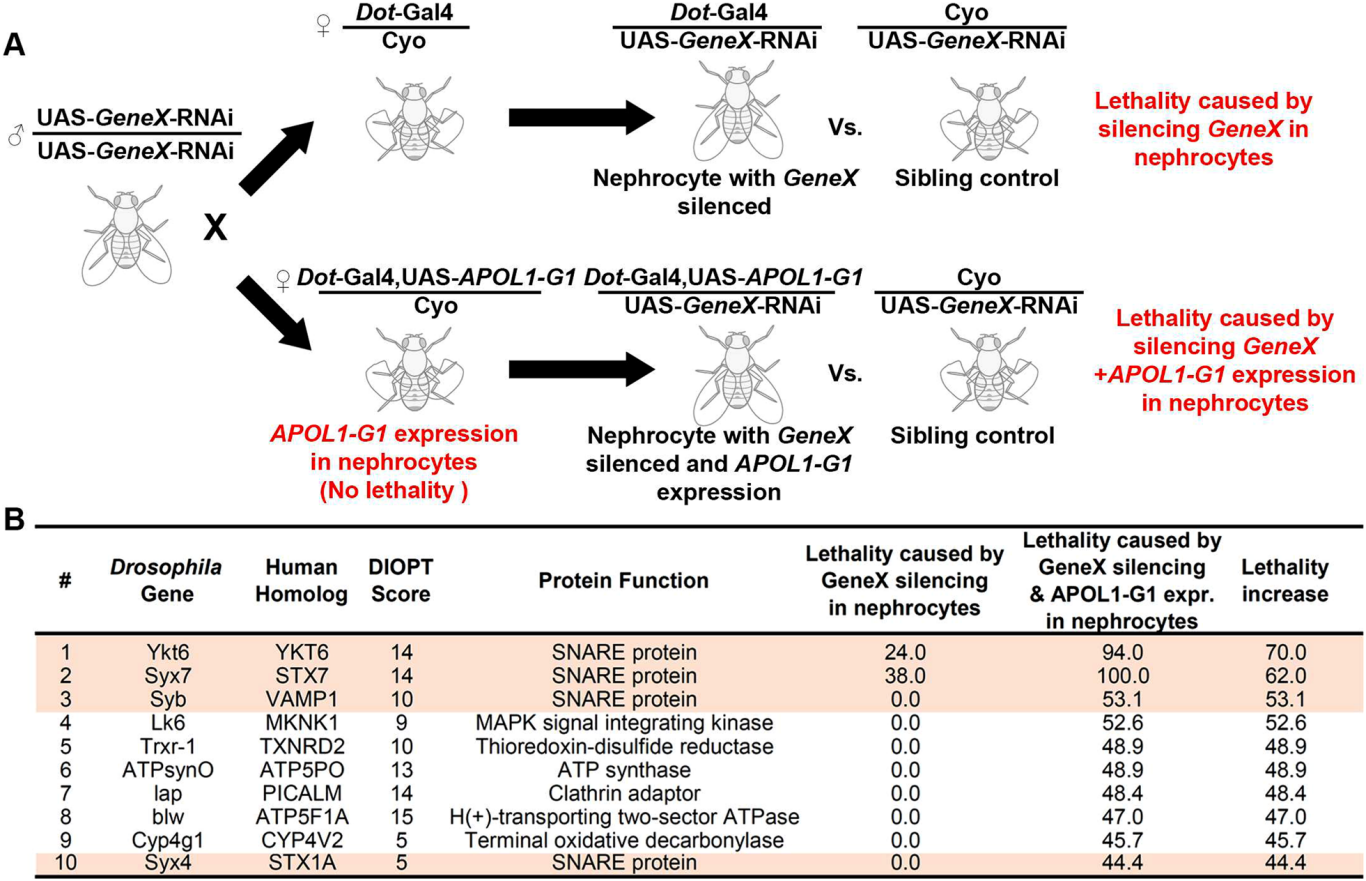
Genetic screen identifies modifiers of *APOL1-G1* induced renal toxicity. **A** Schematic illustration of genetic screen designed to identify genetic modifiers of *APOL1-RA* (*G1*) induced renal toxicity, with adult fly hatching lethality as read-out. *Hand*-GFP, nephrocyte marker; *Mhc*-ANF-RFP, nephrocyte function read-out based on ANF uptake; *Dot*-Gal4/Cyo, nephrocyte-specific driver with CyO balancer chromosome; UAS-*APOL1-G1*, *APOL1-G1* transgene; UAS-GeneX-RNAi, UAS-based RNAi transgenic lines. **B** Top 10 genes found to increase adult fly hatching lethality caused by *APOL1-G1* compared to lethality observed for *APOL1-G1* alone (~ 5%; not shown) or GeneX-RNAi by itself. Genes encoding SNARE proteins have been highlighted. DIOPT, DRSC Integrative Ortholog Prediction Tool

Next, we carried out an unbiased genetic modifier screen designed to rapidly detect any fly gene that might modulate APOL1’s toxicity in nephrocytes. We used a nephrocyte-specific driver (*Dot*-Gal4) and a curly winged (CyO) balancer chromosome (preventing mutation loss in the population and allelic separation by meiotic recombination), with or without the *APOL1-G1* transgene (UAS-*APOL1-G1*). These flies were then crossed to thousands of UAS-based RNAi transgenic lines (UAS-GeneX-RNAi, where GeneX is the gene of interest), thus enabling nephrocyte-specific silencing (Fig. 2A). Straight winged progeny carry nephrocyte *Dot*-Gal4 driven GeneX-RNAi, while curly winged (CyO) flies only carry the GeneX-RNAi which is not expressed in nephrocytes without the Gal4 driver. Thus, non-modifying genes generate 1:1 straight:curly winged ratio offspring. *Dot*-Gal4 driven *APOL1-G1* by itself did not cause lethality at adult hatching (lethality 0.0; data not shown). Rarely did silencing GeneX by itself cause lethalty (e.g., HSC70-4, 61.7% lethality; data not shown); most genes showed little (< 5%) or no lethality at adult hatching. We selected genes that when silenced caused the biggest difference in adult hatching rate on an *APOL1-G1* background compared to the control background (i.e., GeneX-RNAi by itself).

We started the genetic modifier screen with the top ~ 2000 genes most highly expressed in the nephrocytes (based on in-house RNA-seq data from nephrocytes dissected from young adult flies; *Drosophila* has > 13,000 protein-encoding genes); reasoning that to modify APOL1-G1 toxicity, the gene/protein would have to be expressed in nephrocytes. The screen identified ~ 100 genes that, when silenced in the nephrocyte, significantly increased lethality before adult fly hatching in an *APOL1-G1* background compared to their control background (Fig. 2B lists the top 10 hits). Surprisingly, the top three hits (*Ykt6*, *Syx7*, and *Syb*) all encoded SNARE proteins. One additional SNARE gene (*Syx4*) was among the top ten candidates (Fig. 2B, highlighted), suggesting that SNARE proteins might play important roles in modulating the toxicity of the *APOL1-G1* protein. Notably, all top ten genes are highly conserved from *Drosophila* to humans (DIOPT scores ranging from 5–15; the maximum score is 15) (Fig. 2B).

### Silencing *Syx7*, *Ykt6* or *Syb* increases *APOL1-G1* toxicity in *Drosophila* nephrocytes

**Fig. 3:**
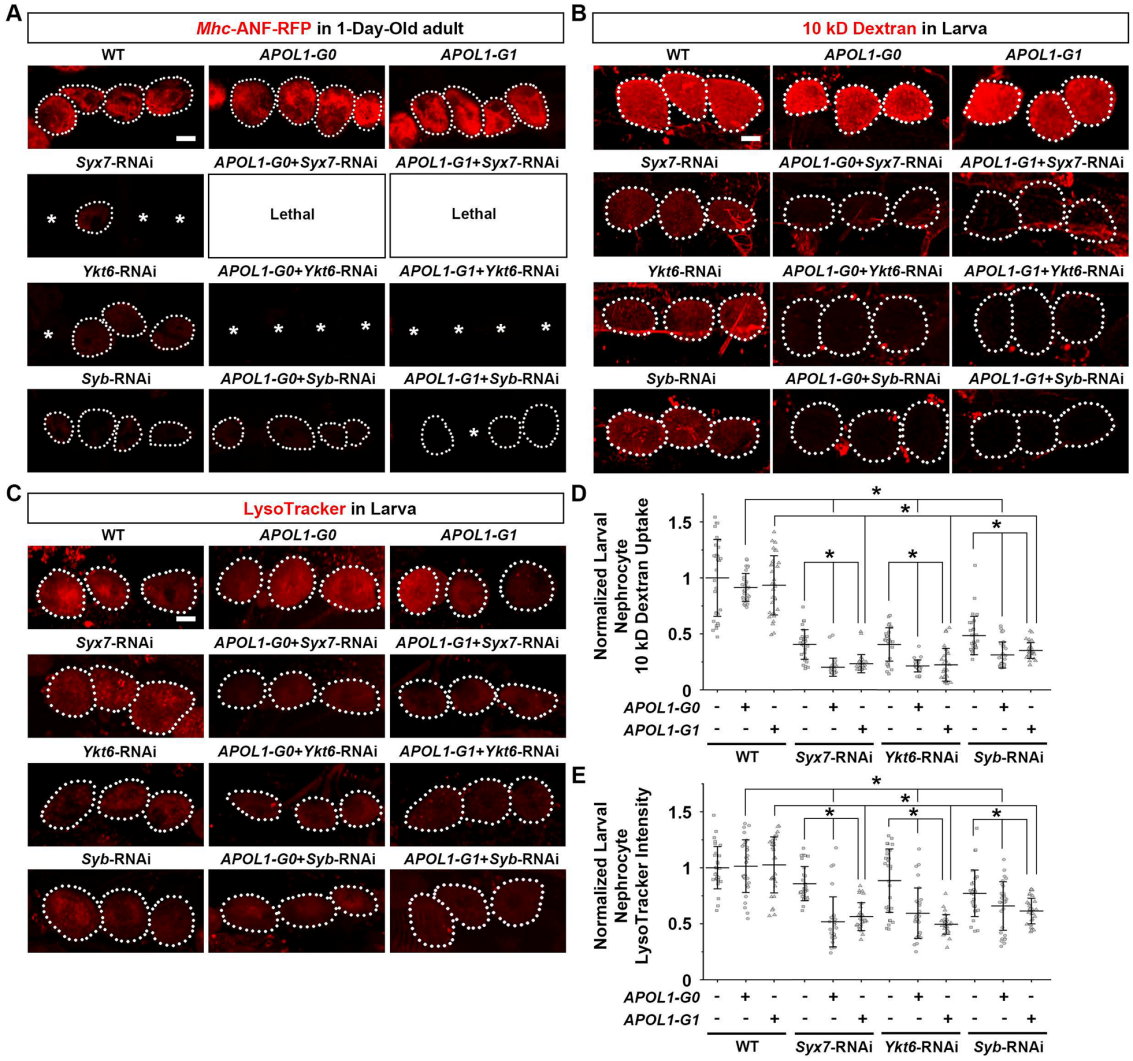
Knockdown of *Syx7*, *Ykt6* or *Syb* facilitates the nephrocyte phenotype caused by *APOL1-G1*. **A** *Mhc*-ANF-RFP uptake (red) by nephrocytes from 1-day-old adult flies using the nephrocyte-specific driver *Dot*-Gal4 to knockdown SNARE protein *Syx7*, *Ykt6* or *Syb* by RNAi construct, either by itself or together with *APOL1-G0* or *APOL1-G1*; carried out at 25 °C. Dotted lines outline individual nephrocytes. Asterisk indicates a missing nephrocyte. **B** Representative images of 10 kD fluorescent dextran particle uptake (red) by nephrocytes from larvae using the nephrocyte-specific driver *Dot*-Gal4 to silence SNARE protein *Syx7*, *Ykt6* or *Syb* by RNAi construct, either by itself or together with *APOL1-G1*; carried out at 25 °C. Dotted lines outline individual nephrocytes. **C** Representative images of LysoTracker (red) by nephrocytes from larvae using nephrocyte-specific driver *Dot-*Gal4 to silence SNARE protein *Syx7*, *Ykt6* or *Syb* by RNAi construct, either by itself or together with *APOL1-G1*; carried out at 25 °C. Dotted lines outline individual nephrocytes. **D** Quantification of 10 kD dextran uptake in **B**, relative to wildtype (WT) values. N = 30 nephrocytes total obtained from 6 larvae. Results represented as mean ± SD, statistical significance (*) P < 0.05. **E** Quantification of LysoTracker intensity in **D**, relative to wildtype (WT) values. N = 30 nephrocytes total obtained from 6 larvae. Results represented as mean ± SD, statistical significance (*) P < 0.05

To determine the extent to which silencing either of these SNARE genes might enhance the *APOL1-G1* phenotype, we examined nephrocytes from newly hatched adult flies. The experiments were carried out at 25 °C (a standard temperature for *Drosophila* crosses at which the APOL1-G0 shows a low level of toxicity). Myosin heavy chain (*Mhc*) promoter-driven atrial natriuretic peptide (ANF)-red fluorescent protein (*Mhc*-ANF-RFP) was secreted into the hemolymph and endocytosed by and visualized in nephrocytes. We compared flies in which one of the SNARE genes (*Syx7*, *Ykt6* or *Syb*) was silenced (RNAi), in the presence and absence (baseline) of an *APOL1-G0* or *APOL1-G1* background (Fig. 3A). At baseline (silenced), *Syx7*, *Ykt6*, and *Syb* showed a reduction in ANF-RFP uptake in nephrocytes, indicating that they are required for nephrocyte function (Fig. 3A). In addition, silencing *Syx7* or *Ykt6* resulted in missing nephrocytes. In the presence of *APOL1-G0* or *APOL1-G1*, these phenotypes were more severe: Silencing *Syx7* led to early lethality without a single adult fly hatching; silencing *Ykt6* led to the death of most nephrocytes in 1-day-old adult flies; and, silencing *Syb* led to reduced nephrocyte numbers in 1-day-old flies (Fig. 3A). Since *APOL1-G1* expression alone does not cause nephrocyte death (or functional loss) in 1-day-old flies—it does at day 20 (Fig. 1)—these findings suggest that silencing *Syx7*, *Ykt6* or *Syb* increased the toxicity caused by *APOL1-G1* expression in nephrocytes.

The notable effects on nephrocytes observed in 1-day-old adult flies after silencing *Syx7*, *Ykt6* or *Syb* alone complicated the study of nephrocyte function and organelle changes when combined with *APOL1-G0* or *APOL1-G1* expression. Therefore, we further examined these interactions at the third-instar larva stage. Unfortunately, the *Mhc*-ANF-RFP transgene does not function at this stage as *Mhc* is expressed in adult fly muscle only [30]; thus, we used an ex vivo functional assay that measures the capacity of dissected nephrocytes to filter and endocytose 10 kD fluorescent dextran particles. *APOL1-G0* or *APOL1-G1* expression alone did not cause nephrocyte functional decline compared with wildtype third instar larvae (Fig. 3B, D). This could be due to APOL1 protein levels not yet having reached a critical threshold at the larval stage. Silencing the SNARE genes *Syx7*, *Ykt6* or *Syb* alone resulted in a reduction of fluorescent dextran uptake compared to wildtype, *APOL1-G0* or *APOL1-G1* fly larvae (Fig. 3B, D), but we observed no changes in the number and size of the nephrocytes at the larval stage (data not shown). Like in the young adult (1-day-old) stage, the functional deficits observed in fly larvae carrying silenced *Syx7*, *Ykt6* or *Syb* were much more pronounced in those that also expressed *APOL1-G0* or *APOL1-G1* (Fig. 3B, D), suggesting that reduced function of these SNARE proteins leads to more severe *APOL1-G0* or *APOL1-G1*-induced nephrocyte toxicity.

Previous studies in human cells [20], fly and yeast [28], and ours in fly [27], have shown that overexpressing *APOL1-G0* and *APOL1-G1* impairs acidification of late endocytic compartments and nephrocyte vacuoles. Therefore, we used a fluorescent LysoTracker dye to test whether silencing of *Syx7*, *Ykt6* or *Syb* would further impair acidification. We examined nephrocyte acidic vacuoles of *APOL1-G0* or *APOL1-G1* third instar larvae and, unlike our previous study in 1, 8 and 15-day adult flies [27], did not find impaired organelle acidification compared to wildtype larvae (Fig. 3C, E). This is in line with the notion that *APOL1* cytotoxicity is dependent on its expression level [20], as the protein levels of APOL1 might be below their critical threshold at the fly larval stage. Silencing *Syx7*, *Ykt6* or *Syb* led to a moderate reduction in LysoTracker fluorescence compared to wildtype flies and those expressing *APOL1-G0* or *APOL1-G1* alone (Fig. 3C, E). However, vesicle acidification was significantly more pronounced in *APOL1-G0* or *APOL1-G1* larvae with concurrent SNARE-RNAi (Fig. 3C, E). These findings indicate that silencing *Syx7*, *Ykt6* or *Syb* encoded SNARE proteins leads to a significant increase in APOL1-G1 nephrocyte toxicity through a further reduction in vesicle acidification.

### Overexpression of *Syx7*, *Ykt6* or *Syb* attenuates *APOL1-G1* toxicity in *Drosophila* nephrocytes

**Fig. 4:**
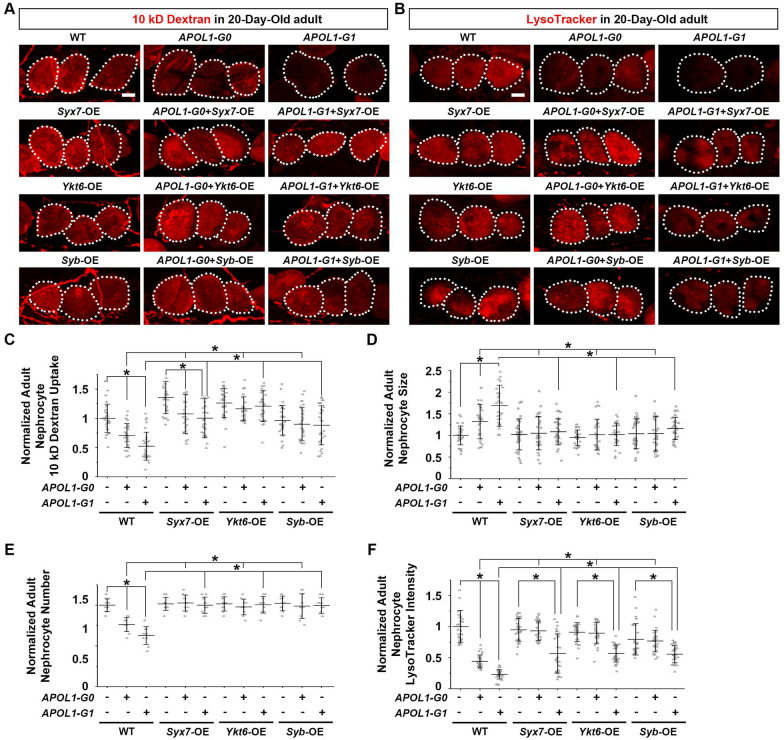
Overexpression of *Syx*7, *Ykt6* or *Syb* attenuates the nephrocyte phenotype induced by *APOL1-G1*. **A** Representative images of 10 kD fluorescent dextran particle uptake (red) by nephrocytes from 20-day-old adult flies using nephrocyte-specific driver *Dot*-Gal4 to overexpress SNARE protein encoding genes *Syx7*, *Ykt6* or *Syb*, either alone or together with *APOL1-G0* or *APOL1-G1*; carried out at 25 °C. Dotted lines outline individual nephrocytes. **B** Representative images of LysoTracker (red) uptake by nephrocytes from 20-day-old adult flies using nephrocyte-specific driver *Dot*-Gal4 to overexpress SNARE protein encoding genes *Syx7*, *Ykt6* or *Syb*, either alone or together with *APOL1-G1*; carried out at 25 °C. *Hand*-GFP transgene expression was visualized as green fluorescence located in nephrocyte nuclei. Dotted lines outline individual nephrocytes. **C** Quantification of 10 kD dextran uptake in **A**, relative to wildtype (WT) values. N = 30 nephrocytes total obtained from 6 flies. Results represented as mean ± SD, statistical significance (*) P < 0.05. **D** Quantification of nephrocyte size in 20-day-old adult flies, relative to WT value. N = 30 nephrocytes total obtained from 6 flies. Results represented as mean ± SD, statistical significance (*) P < 0.05. **E** Quantification of nephrocyte number in 20-day-old adult flies, relative to wildtype (WT) values. N = 10 flies. Results represented as mean ± SD, statistical significance (*) P < 0.05. (**F**) Quantification of LysoTracker intensity in **B**, relative to wildtype (WT) values. N = 30 nephrocytes total obtained from 6 flies. Results represented as mean ± SD, statistical significance (*) P < 0.05

Since silencing *Syx7*, *Ykt6* or *Syb* increased *APOL1-G1*-induced nephrocyte toxicity, we presumed that their overexpression could decrease toxicity. To test this hypothesis, we generated transgenic fly lines that overexpressed the individual SNARE genes (*Syx7*, *Ykt6*, and *Syb*) in the presence or absence of an *APOL1-G1* background. A baseline was established based on the effects of each of these SNARE genes alone. Subsequently, using the 10 kD fluorescent dextran uptake assay, we found that overexpression of *Syx7*, *Ykt6* or *Syb* alone specifically in the nephrocytes did not alter their function, even in 20-day-old flies (Fig. 4A, C). Nor did we detect any changes in nephrocyte size and number, or vesicle acidification in these flies (Fig. 4B, D, E, F). However, overexpressing the SNARE proteins—encoded by *Syx7*, *Ykt6* or *Syb*—in *APOL1-G1* nephrocytes, attenuated various nephrocyte defects in 20-day-old *APOL1-G0* or *APOL1-G1* adult flies. These SNAREs counteracted APOL1-induced adverse changes in nephrocyte function (Fig. 4A, C), size (Fig. 4C), and number (Fig. 4D). Even the reduced acidification caused by *APOL1-G0* or *APOL1-G1* in fly nephrocytes were significantly attenuated by overexpressing *Syx7*, *Ykt6* or *Syb* in these cells (Fig. 4E, F). These results suggest that SNARE proteins can protect nephrocytes from APOL1-induced toxicity.

### APOL1 directly co-localizes and directly interacts with Syx7, Ykt6, and Syb proteins in mammalian kidney cells

**Fig. 5:**
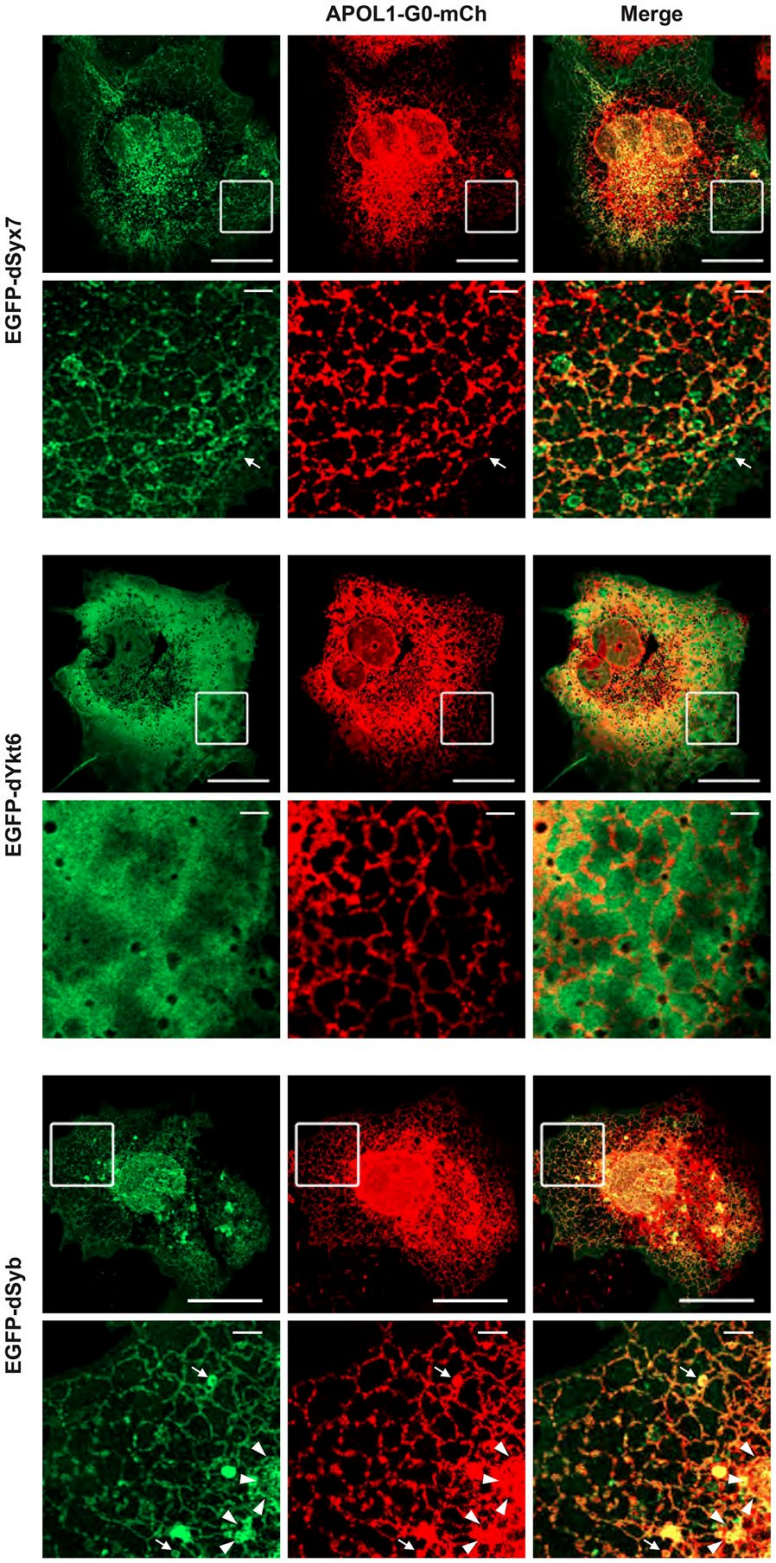
SNARE proteins co-localize with APOL1. Representative images obtained in COS-7 cells transfected with APOL1-G0-mCherry (mCh; red) and EGFP-tagged (green) SNARE protein (dSyx7, dYkt6 or dSyb). The squares outline the magnified images shown in the lower panels. Endoplasmic reticulum (ER) is observed as a typical reticular network formation covering the entire peripheral of the cell (see magnified images); arrows point to endosomes; arrowheads point to Golgi system. Scale bar = 20 μm; (magnification) = 2 μm

Next, we tested whether the protective effects of *Syx7*, *Ykt6* or *Syb* against *APOL1* toxicity involve direct interaction between APOL1 and the SNARE proteins. We examined the cellular distribution of APOL1 and SNARE proteins in COS-7 African green monkey kidney cells, which possess ideal properties for high resolution visualization of intracellular organelles. Double-fluorescent live cell confocal imaging showed that APOL1 protein is localized at vesicular structures such as the endoplasmic reticulum (ER), Golgi complex, and endosomes (Fig. 5). Syx7 and Syb, both type IV single transmembrane proteins known to localize to vesicular structures, showed strong co-distribution with APOL1 protein (Fig. 5). Due to absence of a transmembrane domain, Ykt6 was localized in the cytosol, but still showed partial co-localization with APOL1 protein distribution at vesicular structures (Fig. 5).

**Fig. 6:**
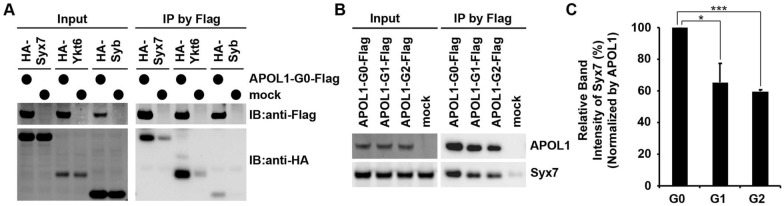
SNARE proteins reduce APOL1-induced cell death in human kidney cells, with efficiency dependent on binding affinity. **A** HEK 293T cells expressing Flag-tagged APOL1-G0 with HA-tagged dSyx7, dYkt6 or dSyb were lysed, and the extracts were immunoprecipitated using anti-Flag M2 affinity gel, followed by immunoblotting with Flag or HA antibody. IB, immunoblotting. **B** HEK 293T cells expressing the indicated Flag-tagged APOL1 variant with HA-tagged dSyx7 were lysed, and the extracts were immunoprecipitated using anti-Flag M2 affinity gel, followed by immunoblotting with Flag antibody. **C** Quantification of the experiments represented in B. Data represented as mean ± SD; *n* = 3 independent experiments. Data represented as mean ± SD. Statistical significance (*) P < 0.05, (***) P < 0.0005

To determine whether co-localization also meant direct interaction between APOL1 and SNARE proteins, we carried out co-immunoprecipitation studies. Since the wildtype APOL1-G0 allele is less toxic than either APOL1-RA allele, we hypothesized a stronger interaction between SNARE proteins and APOL1-G0; thus, we used the APOL1-G0 allele for this assay. We expressed Flag-tagged APOL1-G0 and HA-tagged SNARE proteins (Syx7, Ykt6 or Syb) in the human embryonic kidney cell line HEK 293T. Co-immunoprecipitation experiments demonstrated direct interaction between the APOL1 protein and each of the Syx7, Ykt6, and Syb proteins (Fig. 6A). These data demonstrate that APOL1 protein co-localizes with SNARE proteins in a subset of kidney cell vesicular structures, and that APOL1 directly binds each of the SNARE proteins (Syx7, Ykt6 or Syb).

### *Drosophila* Syx7 protein shows higher binding affinity for APOL1-G0 than either APOL1-G1 or G2 protein

Next, we determined whether SNARE protein binding affinity differed between the non-risk allele APOL1-G0 and the risk alleles APOL1-G1 and APOL1-G2. We focused on *Drosophila* Syx7 because of its complete lethality when expressed with APOL1 (Fig. 2) and its strong co-localization with APOL1 (Fig. 5). Co-immunoprecipitation studies in HEK 293T cells demonstrated that the binding affinity of Syx7 to APOL1-G0 protein was significantly stronger, when compared to its affinity for APOL1-G1 or APOL1-G2 (Fig. 6B, C).

## Discussion

The large-scale unbiased genetic screen in this study exemplifies the unique advantages of *Drosophila* as an efficient in vivo model system to test the effects of APOL1 expression levels on nephrocyte toxicity. Given that both unnatural and different haplotype backgrounds can alter APOL1 cytotoxicity [31], our transgenic flies were generated with *APOL1-G1* cDNA derived from podocytes cultured from a child with HIVAN [32], as described before [27]. The *APOL1-G1* cDNA contains the haplotypes E150, I228 and K255, and differs from *APOL1-G0*—used to generate the control flies—only at S342 and I384 (Fig. 1A). The *APOL1-G2* cDNA contains the *G1* haplotypes (E150; I228; K255) as well as the dual deletion of amino acids N388 and Y389, characteristic of *G2* (Fig. 1A). Therefore, all our transgenic constructs are physiologically relevant and carry common natural haplotypes seen in people of African ancestry who develop APOL1 nephropathies. Furthermore, we reduced the *APOL1-G0* expression in flies to almost below its threshold toxicity range (by lowering the fly environmental temperature to destabilize the Gal4 driver). In this manner, we mitigated the toxicity observed in our previous study in transgenic flies carrying the wildtype APOL1-G0 allele specifically in nephrocytes [27] (Fig. 1); thus, closer mimicking the condition in humans carrying the wildtype/reference alleles.

**Fig. 7:**
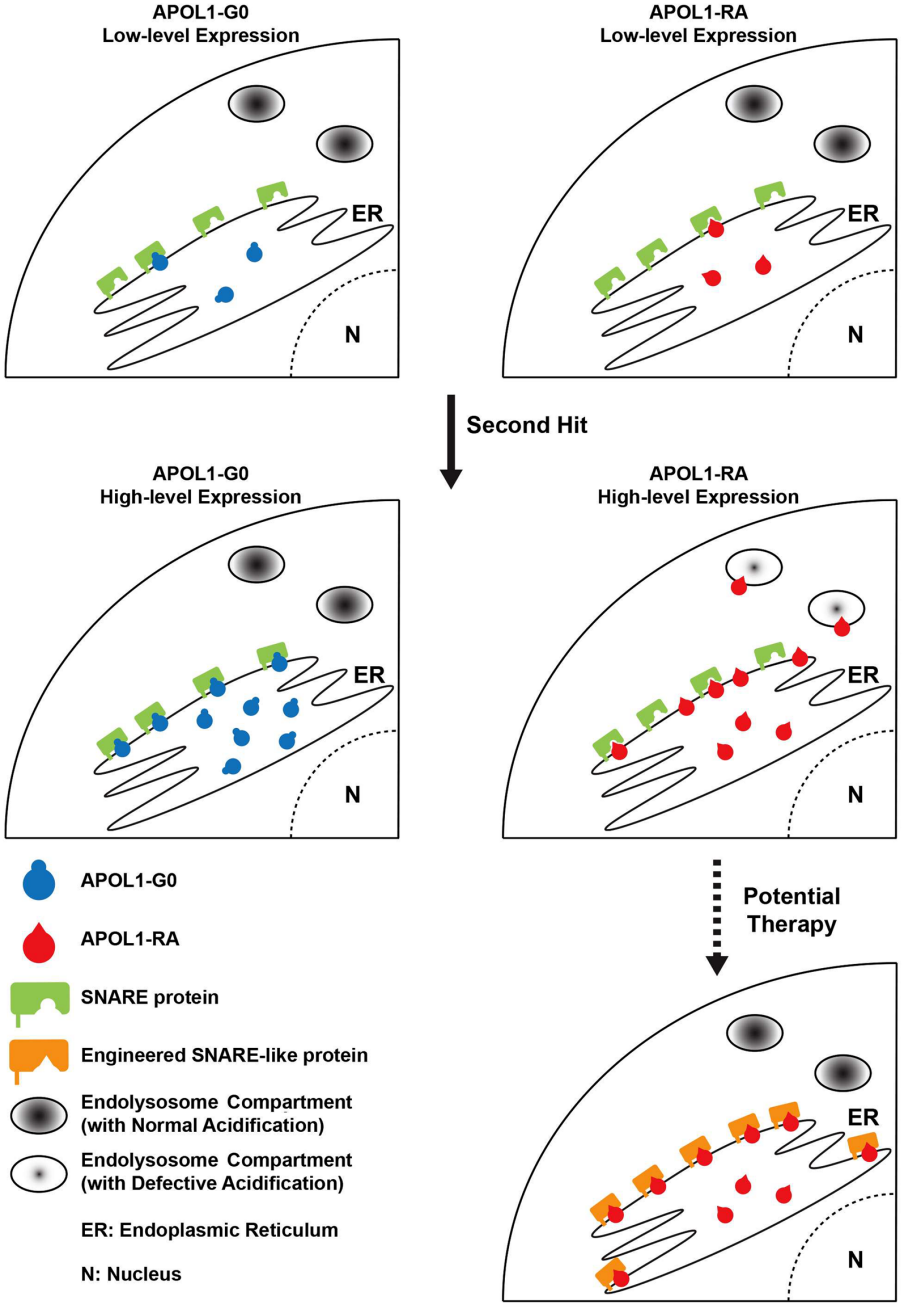
Model of SNARE protective pool to antagonize APOL1-induced toxicity. Schematic representation of the SNARE protective pool model as a strategy to evade APOL1 toxicity. (Left) Under typical conditions, SNARE proteins provide protection against APOL1 toxicity by binding APOL1 protein which localizes APOL1 to the ER and thus prevents it from forming pores in lysosomal membranes. The SNARE proteins in the protective pool effectively bind APOL1-G0 and prevent toxic effects even in the event of a second hit, be it genetic predisposition or environmental. (Right) SNARE protein binding affinity for APOL1 risk alleles (APOL1-RA; APOL1-G1 and APOL1-G2) is reduced, compared to APOL1-G0. Under typical conditions this is still largely sufficient. However, when a second hit occurs, the protective pool cannot offer adequate compensation. Once a certain threshold is breached this results in unbound APOL1-RA, which in turn locates to the lysosome, where it forms pores thereby causing lysosomal acidification and cytotoxicity. (Bottom right) This model implicates expanding the SNARE protective pool as a potential therapeutic strategy. For example, one could overexpress SNARE proteins (as was shown effective for Syx7, Ykt6, and Syb), or a SNARE-like molecule. The increased SNARE protective pool has greater ability to bind and capture APOL1-RA, and to compensate against a second hit. By preventing unbound APOL1-RA to escape and locate at the cell organelles, the enhanced pool can reduce (or even eliminate) APOL1 toxic effects

To study *APOL1*-associated CKD in *Drosophila*, we used a high-throughput readout for adult fly hatching rate; thus, quickly screening thousands of genes—with known expression in nephrocytes based on in house nephrocyte RNA-seq data—for their possible role in modifying APOL1 cytotoxicity. The top three hits (*Syx7, Ykt6*, and *Syb*) encode SNARE proteins, with additional SNARE encoding genes in the top 25 (data not shown). Collectively our data support a novel pathologic paradigm that a selective pool of protective SNARE proteins bind to APOL1 and neutralize its cytotoxicity (Fig. 7). Assuming a two-hit mechanism, we propose that the second hit increases the intracellular levels of APOL1. Subsequently, the risk variants, which have less affinity for binding the pool of protective SNARE proteins, remain free to traffic among the intracellular compartments and induce cytotoxic effects acting on the plasma membrane [18, 33], mitochondria [34–36], trafficking organelles [20, 27, 28], and/or lysosomes [13, 27]. This paradigm is consistent with the notion that APOL1 appears more toxic when it is released from the ER and traffics to the plasma membrane, mitochondria, and/or endolysosomal compartments, where it can induce pores and impair the function of cell trafficking organelles [4, 33, 37]. However, excessive levels of APOL1 when accumulated in the ER, if not neutralized, can trigger the ER stress response [18, 38–40]. Taken together, in this model, APOL1-G0 is less toxic for nephrocytes, at least partially, due to its higher binding affinity to the protective pool of SNARE proteins, which neutralize its cytotoxic activity in endolysosomal compartments. In contrast, APOL1-RA (G1 and G2) have reduced binding affinity to the protective pool of SNARE proteins and thus are more likely to slip through this protective mechanism and target other cellular organelles.

Our model also illustrates how high intracellular levels of APOL1-G0 would overload the pool of protective SNARE proteins, thus resulting in dysfunctional fly nephrocytes [27]. In fact, cultured human podocytes and renal epithelial cells that overexpress wildtype APOL1, display cytotoxicity and promptly die. Therefore, doxycycline-inducible-*APOL1* renal epithelial cell culture model systems have been developed to regulate its expression and cytotoxicity [41, 42]. Presumably, the reduced amount of APOL1-G0 protein in these systems more closely mimics the physiological levels in human renal cells. However, it is difficult to determine the physiological levels of APOL1 expressed in these cell lines, since they also carry the endogenous *APOL1* gene. Nonetheless, we assume that APOL1 might be sufficiently bound by the SNARE protective pool thereby preventing its toxic effects. Overall, this new pathologic paradigm supports the assumption that the risk of developing *APOL1*-associated kidney diseases is a function of the APOL1 expression levels in podocytes; and follows a recessive gain-of-function mode of inheritance [25]. Our data further suggest that the normal function of the protective pool of SNARE proteins also plays a role in the pathogenesis of these kidney diseases. Therefore, any manipulation to increase the protective pool of SNARE proteins, either genetically or through pharmacological intervention, may reduce or even prevent the renal toxicity caused by the *APOL1* risk alleles (Fig. 7). We are pursuing further study into the mechanisms by which SNARE protein rescue APOL1-induced toxicity to include how SNARE and APOL1 might affect each other’s expression, whether reduced SNARE proteins affects ER stress, and whether SNARE protein variants might affect protective abilities.

A recent clinical trial demonstrated that the small molecule drug inaxaplin significantly reduced proteinuria in patients with APOL1-associated focal segmental glomerulosclerosis [9]. Using in vitro and in vivo assays they showed that inaxaplin directly binds the APOL1 protein, thus preventing channel formation, and does so in a dose-dependent manner [9]. It would be interesting to test whether increasing the SNARE protein pool and treatment with inaxaplin complement each other in treating APOL1 nephropathy, as both act by binding APOL1 protein and, we suspect, act through different pathways.

## Conclusions

Using the *Drosophila* model system to conduct a genetic screen for modifiers of APOL1, we identified SNARE proteins, Syx7, Ykt6, and Syb, as antagonists of APOL1-induced cytotoxicity. The human homology of *Drosophila* Syb is VAMP8. This SNARE protein has been shown to directly bind APOL1-G0 and to co-localize with APOL1 protein in human glomerular podocytes; and its binding affinity for APOL1-G0 was significantly higher than for APOL1-G1 or G2 [43]. Notably, of the three SNARE proteins in our study, Syb had the weakest binding affinity for APOL1, suggesting that its role as one of the SNARE protective pool components is likely less effective than that of Syx7 or Ykt6. Further studies are warranted to identify SNARE proteins that play similar protective roles in human podocytes, or mouse models, and to advance the design of SNARE-like (or SRA-like) peptides capable of binding the APOL1-RA proteins as therapeutics to prevent the progression of *APOL1*-associated CKD.

## Methods

### Transgenic *Drosophila* lines

Flies were reared on standard food and temperature (25 °C). The following strains were used in this study: *Hand*-GFP, *Dot*-Gal4, and *Mhc*-ANF-RFP. The UAS-RNAi transgenic lines (TRiP lines) were obtained from the Bloomington Drosophila Stock Center. The UAS-*APOL1-G0*, UAS-*APOL1-G1*, UAS-*Syx7*-OE, UAS-*Ykt6*-OE, and UAS-*Syb*-OE transgenic fly lines were generated in-house.

### *Drosophila* genetic modifier screen

For the genetic modifier screen, we generated *APOL1* transgenic flies by combining a nephrocyte functional read-out (*Mhc*-ANF-RFP), a nephrocyte cell marker (*Hand*-GFP), a nephrocyte-specific driver (*Dot*-Gal4), and the *APOL1-G1* transgene (UAS-*APOL1-G1*) with a CyO balancer chromosome. Female flies with *Dot*-Gal4 driven nephrocyte-specific expression of *APOL1-G1* were then crossed with male transgenic flies carrying a UAS-RNAi gene silencing construct targeting a specific fly gene from the RNAi *Drosophila* library (Fig. 2A). A total of ~ 2000 fly genes were screened for the *APOL1-G1* induced nephrocyte toxicity phenotype, with two independent UAS-RNAi silencing constructs to test each gene. Progeny were initially screened for emergence of straight winged adult flies, indicating that silencing of the given gene by RNAi expression in nephrocytes had prevented or enhanced *APOL1-G1* induced pupal-stage death. Curly-winged adult flies inherited the CyO balancer chromosome rather than the *Dot*-Gal4 driver and therefore expressed neither UAS-*APOL1-G1* nor RNAi in their nephrocytes. We calculated the mortality rate for each transgenic *APOL1-G1*; RNAi line using the formula: %Mortality = [(Curly-Straight)/Curly] × 100. Mortality rates were compared to *APOL1-G1* (~ 5%) and RNAi overexpression alone fly lines. Decreased mortality indicates prevention, and increased mortality rates indicate enhanced *APOL1-G1* induced toxicity. Subsequently, straight-winged adult fly progeny, 20-day-old flies, were further examined for changes in nephrocyte function, number, and size.

### Nephrocyte function (RFP uptake assay), number and size (confocal imaging) in *Drosophila*

The ANF-RFP uptake assay was used to screen nephrocyte function in the *Mhc*-ANF-RFP, *Hand*-GFP, *Dot*-Gal4, UAS-*APOL1-G1*; UAS-RNAi and *Mhc*-ANF-RFP, *Hand*-GFP, *Dot*-Gal4, UAS-*APOL1-G0*; UAS-RNAi transgenic lines. Adult fly nephrocytes were dissected and kept in artificial hemolymph (fly blood), and then fixed in 4% paraformaldehyde in 1X phosphate buffered saline (1XPBS) for 10 min. ANF-RFP uptake by nephrocytes was assayed by fluorescence confocal microscopy (ZEISS LSM 900; see details below). Nephrocyte number and cell size were determined by fluorescent confocal microscopy (ZEISS LSM 900; see details below), then compared to wildtype and *APOL1-G1* transgenic flies without RNAi.

### Dextran uptake assay in *Drosophila* nephrocytes

The dextran uptake assay was used to measure nephrocyte filtration function ex vivo from *APOL1-G0*; UAS-RNAi, *APOL1-G1*; UAS-RNAi, *APOL1-G0*; SNARE gene overexpression and *APOL1-G1*; SNARE gene overexpression transgenic lines. Nephrocytes were dissected from third instar larvae or 20-day-old adult flies and kept in artificial hemolymph. Cells were incubated with AlexaFluor 568-dextran 10 kD (0.05 mg/ml; Invitrogen) for 20 min, and then fixed with 4% paraformaldehyde in 1XPBS for 10 min. Dextran uptake capacity was based on nephrocyte fluorescence levels, assayed by fluorescence confocal microscopy in live cells (ZEISS LSM 900; see details below). For quantification, 30 nephrocytes were analyzed from 6 larvae or female adult flies per genotype. The results have been presented as mean ± SD. Statistical significance (*) has been defined as P < 0.05.

### LysoTracker assay in *Drosophila* nephrocytes

The LysoTracker assay was used to examine acidification of organelles in ex vivo nephrocytes from *APOL1-G0*; UAS-RNAi, *APOL1-G1*; UAS-RNAi, *APOL1-G0*; SNARE gene overexpression and *APOL1-G1*; SNARE gene overexpression transgenic lines. Nephrocytes from adult 20-day-old flies were dissected and kept in artificial hemolymph. Cells were incubated with LysoTracker (Red DND-99; Thermo Fisher Scientific) for 20 min used according to manufacturer instructions. LysoTracker uptake levels were assayed by fluorescence confocal microscopy (ZEISS LSM 900; see details below). Afterwards the cells were fixed with 4% paraformaldehyde in 1XPBS for 10 min to facilitate immunostaining. For quantification, 30 nephrocytes were analyzed from 6 female flies per genotype. The results have been presented as mean ± SD. Statistical significance (*) has been defined as P < 0.05.

### Fluorescent confocal microscopy of *Drosophila* nephrocytes

Confocal imaging was performed with a ZEISS LSM 900 microscope using a 63X Plan-Apochromat 1.4 N.A. oil objective under Airyscan SR mode using ZEN acquisition software (ZEN blue edition, version 3.2; ZEISS). For quantitative comparison of intensities, settings were chosen to avoid oversaturation and applied across images for all samples within an assay. ImageJ Software Version 1.49 was used for image processing.

### Statistical analysis for *Drosophila* assays

Statistical tests were performed using PAST.exe software (http://folk.uio.no/ohammer/past/index.html). Data were first tested for normality using the Shapiro–Wilk test ($\alpha$ = 0.05). Kruskal–Wallis H-test followed by a Dunn’s test for comparisons between multiple groups. Statistical significance was defined as P < 0.05.

### Human and nonhuman primate kidney cell lines, plasmids, and transfection

The HEK 293T and COS-7 cells were purchased from ATCC and their cultures maintained in DMEM medium (Corning cellgro) containing 10% fetal bovine serum and 100 units/ml Penicilin/Streptomycin (Thermo Fisher). Mammalian expression constructs for *APOL1-G0*, *APOL1-G1*, *APOL1-G2*, *dSyx7*, *dYkt6*, and *dSyb* were generated by ligating the PCR-amplified cDNA fragment into pCMV6, pHM6, mCherry2-N1, and pEGFP-C3 for tagging with Flag, HA, mCherry, and EGFP, respectively (plasmids were obtained from Addgene). Transfection was performed with TransIT-293 (Mirus) for HEK 293T cells, and with Lipofectamine 3000 (Invitrogen) for COS-7 cells.

### Airyscan confocal microscopy of kidney cells

To observe the localization of APOL1-G0 and SNARE proteins, COS-7 cells were seeded at 1.5 × 10^5^ per well in a 35 mm dish (ibidi GmbH, Germany) coated with 1 mg/ml fibronectin (Sigma). A total of 2 µg plasmid (1 µg APOL1-G0, 1 µg SNARE) was transfected into cells using Lipofectamine 3000 (Invitrogen) following the manufacturer’s instruction. Live confocal microscopy was performed 16 h post transfection. Cells were washed with 1XPBS and then kept in HEPES buffered no phenol red DMEM medium (ThermoFisher). Cells were imaged with a 63X objective on an LSM 900 confocal microscope equipped with an Airyscan detector array using ZEN acquisition software (ZEN blue edition, version 3.2; ZEISS).

### Immunoprecipitation and immunoblotting from kidney cell samples

For Fig. 6, HEK 293T cells were co-transfected with 100 ng of empty vector or APOL1 variant plasmids, together with 900 ng of SNARE plasmids. The HEK 293T cells were harvested 16 h post-transfection and lysed in the Nonidet P-40 lysis buffer containing 50 mM Tris–HCl pH 7.4, 150 mM sodium chloride, 2 mM magnesium chloride, 0.5% Nonidet P-40, and a protease inhibitor mixture. After centrifugation at 16,100×*g* for 10 min to remove insoluble material, the cleared cell lysates were subjected to immunoprecipitation using an anti-Flag M2 affinity gel (Sigma). Following, immunoblotting was performed according to standard protocol. APOL1 proteins were Flag-tagged, while SNARE proteins carried HA-tags. HRP-conjugated secondary antibodies (Anti-Rabbit IgG–Peroxidase antibody, Sigma #A6154; Anti-Mouse IgG-Peroxidase antibody, Sigma #A4416; used at 1:2,000 dilution) or fluorescence-labeled secondary antibodies (IRDye 680RD Goat anti-Mouse IgG, LI-COR #926–68070; IRDye 800CW Goat anti-Rabbit IgG, LI-COR #926–32211; used at 1:5000 dilution) were used for detection. HRP signal was detected by the enhanced chemiluminescence method (ECL) and recorded by a Chemidoc Touch Imaging System (Bio-Rad). The intensities of the detected HRP protein bands were quantified using Image Lab software (Bio-Rad). The fluorescent bands were imaged and quantified by a LI-COR Odyssey infrared imager using the software (Image Studio Version 5.2) provided by the manufacturer (LI-COR).

### Statistical analysis for in vitro human/primate assays

All experiments were repeated at least twice with a representative gel and combined graph shown in the figure. The *n* value in the figure legend indicates the number of independent experiments conducted. Error bars show the mean ± SD. Data were first tested for normality by using the Shapiro–Wilk test (a = 0.05). Kruskal–Wallis H-test followed by a Dunn’s test was used for comparisons between multiple groups (non-normal distributed data). P values < 0.05 were considered significant.

## Data Availability

The materials and datasets used and/or analyzed during the current study are available from the corresponding authors on reasonable request.

## References

[1] Genovese G, Friedman DJ, Ross MD, Lecordier L, Uzureau P, Freedman BI (2010). Association of trypanolytic ApoL1 variants with kidney disease in African Americans. Science.

[2] Genovese G, Tonna SJ, Knob AU, Appel GB, Katz A, Bernhardy AJ (2010). A risk allele for focal segmental glomerulosclerosis in African Americans is located within a region containing APOL1 and MYH9. Kidney Int.

[3] Tzur S, Rosset S, Shemer R, Yudkovsky G, Selig S, Tarekegn A (2010). Missense mutations in the APOL1 gene are highly associated with end stage kidney disease risk previously attributed to the MYH9 gene. Hum Genet.

[4] Vanhamme L, Paturiaux-Hanocq F, Poelvoorde P, Nolan DP, Lins L, Van Den Abbeele J (2003). Apolipoprotein L-I is the trypanosome lytic factor of human serum. Nature.

[5] Freedman BI, Kopp JB, Langefeld CD, Genovese G, Friedman DJ, Nelson GW (2010). The apolipoprotein L1 (APOL1) gene and nondiabetic nephropathy in African Americans. J Am Soc Nephrol.

[6] Ashley-Koch AE, Okocha EC, Garrett ME, Soldano K, De Castro LM, Jonassaint JC (2011). MYH9 and APOL1 are both associated with sickle cell disease nephropathy. Br J Haematol.

[7] Larsen CP, Beggs ML, Saeed M, Walker PD (2013). Apolipoprotein L1 risk variants associate with systemic lupus erythematosus-associated collapsing glomerulopathy. J Am Soc Nephrol.

[8] Freedman BI, Langefeld CD, Andringa KK, Croker JA, Williams AH, Garner NE (2014). End-stage renal disease in African Americans with lupus nephritis is associated with APOL1. Arthritis Rheumatol.

[9] Egbuna O, Zimmerman B, Manos G, Fortier A, Chirieac MC, Dakin LA (2023). Inaxaplin for proteinuric kidney disease in persons with two APOL1 variants. N Engl J Med.

[10] Reeves-Daniel AM, DePalma JA, Bleyer AJ, Rocco MV, Murea M, Adams PL (2011). The APOL1 gene and allograft survival after kidney transplantation. Am J Transplant.

[11] Freedman BI, Locke JE, Reeves-Daniel AM, Julian BA (2017). Apolipoprotein L1 gene effects on kidney transplantation. Semin Nephrol.

[12] Doshi MD, Ortigosa-Goggins M, Garg AX, Li L, Poggio ED, Winkler CA (2018). APOL1 genotype and renal function of black living donors. J Am Soc Nephrol.

[13] Lan X, Jhaveri A, Cheng K, Wen H, Saleem MA, Mathieson PW (2014). APOL1 risk variants enhance podocyte necrosis through compromising lysosomal membrane permeability. Am J Physiol Renal Physiol.

[14] Cheng D, Weckerle A, Yu Y, Ma L, Zhu X, Murea M (2015). Biogenesis and cytotoxicity of APOL1 renal risk variant proteins in hepatocytes and hepatoma cells. J Lipid Res.

[15] Lan X, Wen H, Lederman R, Malhotra A, Mikulak J, Popik W (2015). Protein domains of APOL1 and its risk variants. Exp Mol Pathol.

[16] Lan X, Wen H, Saleem MA, Mikulak J, Malhotra A, Skorecki K (2015). Vascular smooth muscle cells contribute to APOL1-induced podocyte injury in HIV milieu. Exp Mol Pathol.

[17] Khatua AK, Cheatham AM, Kruzel ED, Singhal PC, Skorecki K, Popik W (2015). Exon 4-encoded sequence is a major determinant of cytotoxicity of apolipoprotein L1. Am J Physiol Cell Physiol.

[18] Olabisi OA, Zhang J-Y, VerPlank L, Zahler N, DiBartolo S, Heneghan JF (2016). APOL1 kidney disease risk variants cause cytotoxicity by depleting cellular potassium and inducing stress-activated protein kinases. Proc Natl Acad Sci U S A.

[19] Bruggeman LA, Wu Z, Luo L, Madhavan SM, Konieczkowski M, Drawz PE (2016). APOL1-G0 or APOL1-G2 transgenic models develop preeclampsia but not kidney disease. J Am Soc Nephrol.

[20] Beckerman P, Bi-Karchin J, Park ASD, Qiu C, Dummer PD, Soomro I (2017). Transgenic expression of human APOL1 risk variants in podocytes induces kidney disease in mice. Nat Med.

[21] Kumar V, Vashistha H, Lan X, Chandel N, Ayasolla K, Shoshtari SSM (2018). Role of apolipoprotein L1 in human parietal epithelial cell transition. Am J Pathol.

[22] Okamoto K, Rausch JW, Wakashin H, Fu Y, Chung J-Y, Dummer PD (2018). APOL1 risk allele RNA contributes to renal toxicity by activating protein kinase R. Commun Biol.

[23] Aghajan M, Booten SL, Althage M, Hart CE, Ericsson A, Maxvall I (2019). Antisense oligonucleotide treatment ameliorates IFN-γ-induced proteinuria in APOL1-transgenic mice. JCI Insight.

[24] Wakashin H, Heymann J, Roshanravan H, Daneshpajouhnejad P, Rosenberg A, Shin MK (2020). APOL1 renal risk variants exacerbate podocyte injury by increasing inflammatory stress. BMC Nephrol.

[25] McCarthy GM, Blasio A, Donovan OG, Schaller LB, Bock-Hughes A, Magraner JM (2021). Recessive, gain-of-function toxicity in an APOL1 BAC transgenic mouse model mirrors human APOL1 kidney disease. Dis Model Mech.

[26] Yoshida T, Latt KZ, Heymann J, Kopp JB (2021). Lessons from APOL1 animal models. Front Med.

[27] Fu Y, Zhu J-Y, Richman A, Zhang Y, Xie X, Das JR (2017). APOL1-G1 in nephrocytes induces hypertrophy and accelerates cell death. J Am Soc Nephrol.

[28] Kruzel-Davila E, Shemer R, Ofir A, Bavli-Kertselli I, Darlyuk-Saadon I, Oren-Giladi P (2017). APOL1-mediated cell injury involves disruption of conserved trafficking processes. J Am Soc Nephrol.

[29] Weavers H, Prieto-Sánchez S, Grawe F, Garcia-López A, Artero R, Wilsch-Bräuninger M (2009). The insect nephrocyte is a podocyte-like cell with a filtration slit diaphragm. Nature.

[30] Zhang F, Zhao Y, Han Z (2013). An in vivo functional analysis system for renal gene discovery in *Drosophila* pericardial nephrocytes. J Am Soc Nephrol.

[31] Lannon H, Shah SS, Dias L, Blackler D, Alper SL, Pollak MR (2019). Apolipoprotein L1 (APOL1) risk variant toxicity depends on the haplotype background. Kidney Int.

[32] Xie X, Colberg-Poley AM, Das JR, Li J, Zhang A, Tang P (2014). The basic domain of HIV-tat transactivating protein is essential for its targeting to lipid rafts and regulating fibroblast growth factor-2 signaling in podocytes isolated from children with HIV-1-associated nephropathy. J Am Soc Nephrol.

[33] Giovinazzo JA, Thomson RP, Khalizova N, Zager PJ, Malani N, Rodriguez-Boulan E (2020). Apolipoprotein L-1 renal risk variants form active channels at the plasma membrane driving cytotoxicity. Elife.

[34] Granado D, Müller D, Krausel V, Kruzel-Davila E, Schuberth C, Eschborn M (2017). Intracellular APOL1 risk variants cause cytotoxicity accompanied by energy depletion. J Am Soc Nephrol.

[35] Ma L, Chou JW, Snipes JA, Bharadwaj MS, Craddock AL, Cheng D (2017). APOL1 renal-risk variants induce mitochondrial dysfunction. J Am Soc Nephrol.

[36] Shah SS, Lannon H, Dias L, Zhang J-Y, Alper SL, Pollak MR (2019). APOL1 kidney risk variants induce cell death via mitochondrial translocation and opening of the mitochondrial permeability transition pore. J Am Soc Nephrol.

[37] Pérez-Morga D, Vanhollebeke B, Paturiaux-Hanocq F, Nolan DP, Lins L, Homblé F (2005). Apolipoprotein L-I promotes trypanosome lysis by forming pores in lysosomal membranes. Science.

[38] Wen H, Kumar V, Lan X, Shoshtari SSM, Eng JM, Zhou X (2018). Biosci Rep.

[39] Gerstner L, Chen M, Kampf LL, Milosavljevic J, Lang K, Schneider R (2022). Inhibition of endoplasmic reticulum stress signaling rescues cytotoxicity of human apolipoprotein-L1 risk variants in *Drosophila*. Kidney Int.

[40] Kruzel-Davila E, Bavli-Kertselli I, Ofir A, Cheatham AM, Shemer R, Zaknoun E (2022). Endoplasmic reticulum-translocation is essential for APOL1 cellular toxicity. iScience.

[41] O’Toole JF, Schilling W, Kunze D, Madhavan SM, Konieczkowski M, Gu Y (2018). ApoL1 overexpression drives variant-independent cytotoxicity. J Am Soc Nephrol.

[42] Datta S, Kataria R, Zhang J-Y, Moore S, Petitpas K, Mohamed A (2020). Kidney disease-associated APOL1 variants have dose-dependent, dominant toxic gain-of-function. J Am Soc Nephrol.

[43] Madhavan SM, O’Toole JF, Konieczkowski M, Barisoni L, Thomas DB, Ganesan S (2017). APOL1 variants change C-terminal conformational dynamics and binding to SNARE protein VAMP8. JCI Insight.

